# A retrospective study of variations in the kinds of diseases discharged from the Department of Infectious Diseases of a large general hospital in Central China during 2013–2019

**DOI:** 10.3389/fpubh.2024.1289972

**Published:** 2024-02-14

**Authors:** Pian Ye, Lei Zhao, Ran Pang, Xin Zheng

**Affiliations:** Department of Infectious Diseases, Union Hospital, Tongji Medical College, Huazhong University of Science and Technology, Wuhan, Hubei Province, China

**Keywords:** general hospital, hepatobiliary diseases, infectious diseases, non-communicable infectious diseases (NCIDs), communicable infectious diseases (CIDs), kinds of diseases, constituent ratio

## Abstract

**Objective:**

To analyze the changing trend of the absolute number and constituent ratio of various in-patient diseases in the Department of Infectious Diseases of a large general hospital in Central China during 2013–2019.

**Methods:**

A retrospective study was conducted to analyze the diagnostic data of discharged patients for seven consecutive years, from 2013 to 2019. The first discharge diagnosis is used as the basis for the disease classification. The absolute number, constituent ratio, and changing trend of major diseases in hepatobiliary diseases and infectious diseases were analyzed.

**Results:**

The changing trend of the diseases during 2013–2019 showed that the absolute number of cases of hepatobiliary disease did not change significantly (*p* = 0.615), while the constituent ratio decreased significantly, from 68.01% in 2013 to 55.29% in 2019 (*p*<0.001). The absolute number (constituent ratio) of cases of infectious diseases increased significantly from 585 (21.91%) in 2013 to 1,244 (36.86%) in 2019 (*p* = 0.015, *p*<0.001). The major part of the increase was non-communicable infectious diseases (NCIDs).

**Conclusion:**

During 2013–2019, the proportion of cases of hepatobiliary disease gradually decreased. The absolute number and proportion of cases of infectious diseases, especially NCIDs, have increased rapidly.

## Introduction

In recent years, with the rapid growth of our economy, the remarkable improvement of sanitary conditions, and the rapid progress of medical science, the large-scale outbreak and prevalence of communicable diseases have been significantly reduced, most communicable diseases have been basically or effectively controlled, and the overall morbidity and mortality of communicable diseases have been significantly reduced (1). The human disease pedigree has obviously changed. With this major change, discipline of communicable diseases is transitioning towards discipline of infectious diseases (including communicable infectious diseases (CIDs) and NCIDs) (2), which are more extensive in scope, richer in connotation, and more complex in structure. Therefore, in recent years, the Department of Infectious Diseases in general hospitals across China has completed the transformation from “Department of Communicable Diseases” to “Department of Infectious Diseases” in terms of name (2, 3). Theoretically, infectious diseases include infections caused by any kind of pathogen, involving all clinical departments of the hospital. For many years, the Department of Infectious Diseases in general hospitals in China has mainly treated hepatobiliary diseases, especially liver diseases (4). At the same time, since infectious diseases have long been the leading cause of fever of unknown origin (FUO), the Department of Infectious Diseases usually also takes on the diagnosis and treatment of febrile patients (5). Therefore, for a long time, the Department of Infectious Diseases of general hospitals in China spontaneously divided the diseases of the department into hepatobilliary dieseases and other infectious diseases. The Department of Infectious Diseases of Union Hospital affiliated with Huazhong University of Science and Technology is the Department of Infectious Diseases of a large general hospital directly under the National Health Commission. Founded in 1950, it treats all kinds of fevers, hepatobiliary diseases, and infectious diseases.

In the late 1990s and early 20th century, according to the needs of the development of the discipline, the Department of Infectious Diseases of Union Hospital was also renamed from “Department of Communicable Diseases” to “Department of Infectious Diseases.” However, it is still unclear whether the absolute number and proportion of cases of various diseases treated in the department have changed after the name change and what the trend is. Therefore, this study retrospectively analyzed the changing trend of the disease spectrum of discharged cases from the Department of Infectious Diseases of Union Hospital affiliated to Huazhong University of Science and Technology in 7 years (2013–2019) so as to understand the diagnosis and treatment status of the Department of Infectious Diseases and provide data support and ideas for the transformation and development of the Department of Infectious Diseases in general hospitals.

## Data and methods

### Objects of study

The discharged cases from the Department of Infectious Diseases during the seven consecutive years from 2013 to 2019 were taken as the objects of study, and the specific diagnostic data of discharged cases were obtained by extracting medical record system information from the Department of Statistics of the hospital. The first discharge diagnosis is used as the basis for the disease classification of discharged cases.

### Research methods

A retrospective investigation was carried out. Based on the first diagnosis of discharged cases, the cases were divided into six categories: hepatobiliary diseases, infectious diseases (except viral hepatitis), neoplastic diseases (except PLCa), rheumatic connective tissue diseases (RCTDs), FUO, and other diseases. According to “Law of the People’s Republic of China on prevention and control of communicable diseases”, we classified infectious diseases included in the law as CIDs, including category A communicable diseases: plague, cholera; category B communicable diseases:Monkeypox, novel coronavirus pneumonia, SARS, AIDS, poliomyelitis, human infection with highly pathogenic avian influenza, measles, epidemic haemorrhagic fever, rabies, Japanese encephalitis, dengue fever, anthrax, bacillary dysentery and amebic dysentery, tuberculosis, typhoid and paratyphoid fever, epidemic cerebrospinal meningitis, whooping cough, diphtheria, newborns Tetanus, scarlet fever, brucellosis, gonorrhea, syphilis, leptospirosis, schistosomiasis, malaria, human infection with H7N9 avian influenza; category C communicable diseases: influenza, mumps, rubella, acute hemorrhagic conjunctivitis, leprosy, epidemic typhus and endemic typhus, kala-azar, echinococcosis, filariasis, infectious diarrhoeal diseases other than cholera, bacterial and amebic dysentery, typhoid and paratyphoid, hand、foot and mouth diseases. Other infectious diseases not covered by the law are classified as NCIDs. The number of each type of case was counted annually, and the constituent ratio of each type of case in each year was calculated. The changes in disease spectrum and clinical characteristics of discharged cases from 2013 to 2019 were retrospectively analyzed, and the clinical characteristics of some common hepatobiliary diseases and infectious diseases were summarized and analyzed.

### Statistical analysis

SPSS 26.0 statistical software was used for numerical analysis. The trend χ2 test was used to compare the number or constituent ratio among different samples. *p* < 0.05 was considered statistically significant.

## Results

### Historical changing trend of the constituent ratio of different infectious diseases over the years

From January 1, 2013, to December 31, 2019, a total of 21,150 cases were discharged from the Department of Infectious Diseases, Union Hospital affiliated with Huazhong University of Science and Technology for 7 consecutive years. The number of discharged cases of various diseases and their proportion to the total number of discharged cases during the same period are shown in Table 1. From 2013 to 2019, the discharged cases in the Department of Infectious Diseases of Union Hospital mainly fell into six categories: hepatobiliary diseases, infectious diseases (except viral hepatitis), neoplastic diseases (except primary liver cancer (PLCa)), RCTDs, FUO, and other diseases. Among them, hepatobiliary diseases accounted for the highest proportion of the total number of discharged cases, followed by infectious diseases (except viral hepatitis). Infectious diseases (other than viral hepatitis) can also be classified as NCIDs and CIDs (Table 1). The third is neoplastic disease (except PLCa).

**Table 1:** The number of discharge cases of various diseases in the Department of Infectious Diseases from 2013 to 2019 and their proportion in the total number of discharge cases (cases, %).

Disease categories	2013	2014	2015	2016	2017	2018	2019
Hepatobiliary diseases	1787 (66.93)	1722 (62.44)	1820 (59.95)	1745 (55.91)	1,501 (51.78)	1709 (51.91)	1806 (53.60%)
Infectious diseases	585 (21.91)	717 (26.00)	871 (28.69)	1,014 (32.49)	1,054 (36.36)	1,183 (35.94)	1,244 (36.86)
NCIDs	338 (12.66)	415 (15.05)	521 (17.16)	610 (19.55)	701 (24.18)	847 (25.73)	902 (26.73)
CIDs	247 (9.25)	302 (10.95)	350 (11.53)	404 (12.94)	353 (12.18)	336 (10.21)	342 (10.13)
Neoplastic diseases	72 (2.70)	86 (3.12)	70 (2.31)	61 (1.95)	67 (2.31)	101 (3.07)	104 (3.08)
RCTDs	36 (1.35)	54 (1.96)	38 (1.25)	49 (1.57)	44 (1.52)	56 (1.70)	64 (1.90)
FUO	129 (4.83)	111 (4.02)	134 (4.41)	130 (4.17)	82 (2.83)	100 (3.04)	45 (1.33)
Other diseases	74 (2.77)	69 (2.50)	77 (2.54)	87 (2.79)	61 (2.10)	87 (2.64)	81 (2.40)
Total number of cases	2,670	2,757	3,036	3,121	2,899	3,292	3,375

Hepatobiliary diseases described in the table include viral hepatitis, PLCa, DILI, AILDs, ALD, CSLD, bacterial hepatitis, HMLDs, NAFLD, NHVH, and other hepatobiliary diseases. Infectious diseases (other than viral hepatitis) include NCIDs and CIDs. NCIDs include infections of respiratory tract, bloodstream, digestive system, urinary tract, skin and soft tissue, infectious endocarditis, bone and joint, nervous system, other sites, and unknown sites. CIDs include SFTS, TB, HFRS, salmonella infection, brucellosis, infectious mononucleosis, bacillary dysentery, malaria, dengue fever, AIDS, and skamushi disease. Neoplastic diseases include malignancies of other organs except PLCa and malignancies of the hematological system. Other cases include cardiovascular diseases, endocrine diseases, other hematological diseases, etc.

**Figure 1:**
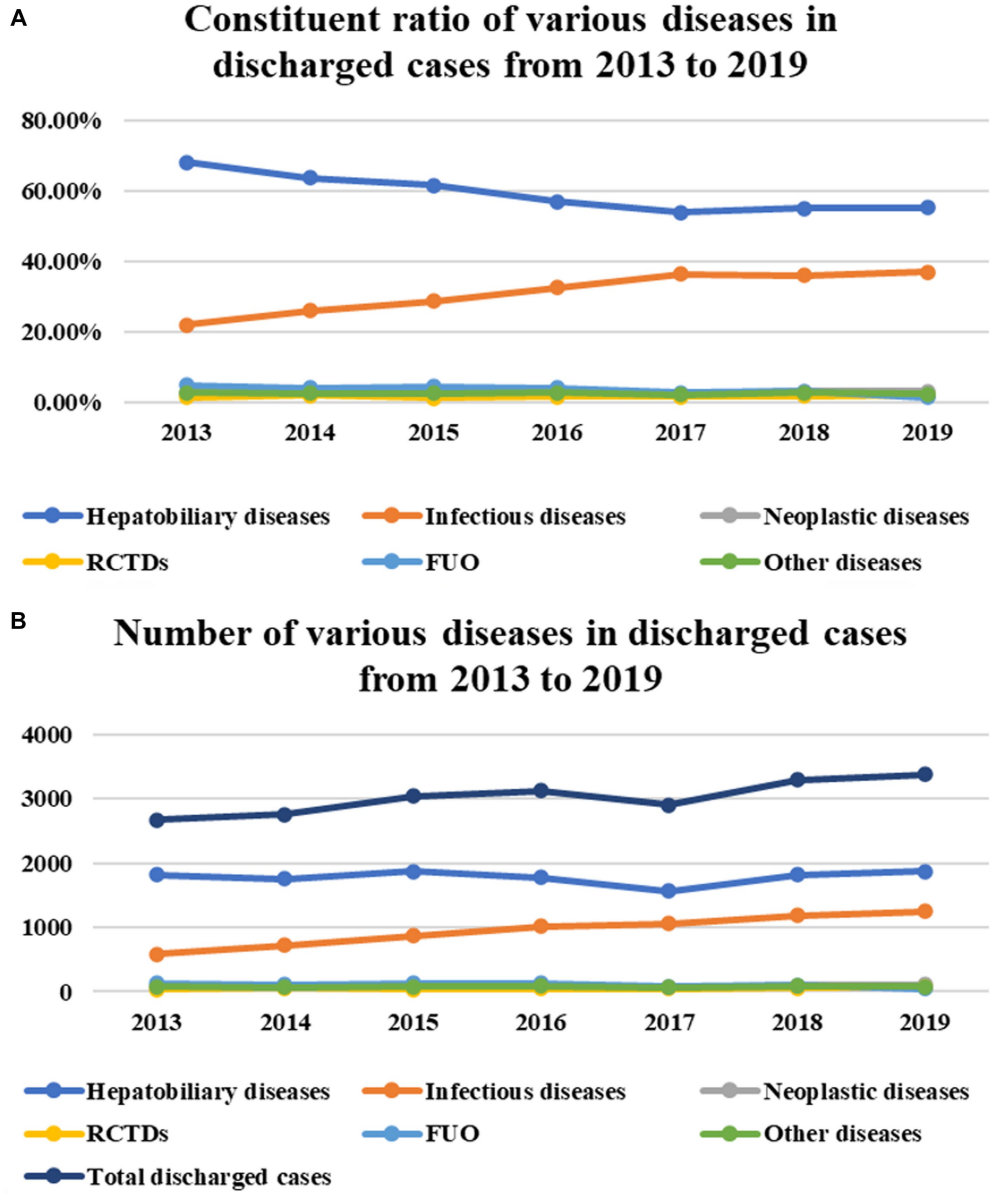
From 2013 to 2019, the number of discharged cases in the Department of Infectious diseases increased year by year for seven consecutive years (*P* < 0.029) **(B)**.The time trend of the absolute number and constituent ratio of each type of disease over the past 7 years showed that the absolute number of cases of hepatobiliary diseases did not change significantly (*P* = 0.615) **(B)**, while the constituent ratio decreased significantly (*P* < 0.001) **(A)**. The absolute number of infectious diseases (excluding viral hepatitis) increased significantly (*P*=0.015) **(B)**. The constituent ratio also increased year by year (*P* < 0.001) **(A)**. In the past 7 years, the absolute number/constituent ratio of cases of neoplastic diseases, RCTDs, and other diseases has not changed significantly (*P*= 0.173, *P* = 0.419; *P*= 0.079, *P* = 0.355; *P* = 0.416, *P* = 0.113) **(A,B)**. The constituent ratio of cases with FUO as the first discharge diagnosis decreased during the 7 years (*P* < 0.001) **(A)**, but the absolute number did not decrease significantly (*P* = 0.056) **(B)**.

The number of discharged cases in the Department of Infectious Diseases during the seven consecutive years from 2013 to 2019 and among which the number of cases of hepatobiliary diseases (constituent ratio) as well as the number of cases (constituent ratio) of infectious diseases (except viral hepatitis) were shown in Table 1. The time trend of the absolute number and constituent ratio of each type of disease over the past 7 years showed that the absolute number of cases of hepatobiliary diseases did not change significantly (*p* = 0.615), while the constituent ratio decreased significantly, from 66.93% in 2013 to 53.60% in 2019 (*p*<0.001). The absolute number of infectious diseases (excluding viral hepatitis) increased significantly from 585 cases in 2013 to 1,244 cases in 2019 (*p* = 0.015). The constituent ratio also increased year by year, from 21.91% in 2013 to 36.86% in 2019 (*p* = 0.001) (Table 1; Figures 1A,B).

In the past 7 years, the absolute number/ constituent ratio of cases of neoplastic diseases, RCTDs, and other diseases has not changed significantly, as shown in Table 1 (*p* = 0.173, *p* = 0.419; *p* = 0.079, *p* = 0.355; *p* = 0.416, *p* = 0.113). Also in Table 1, we can see the absolute number and constituent ratio of cases with FUO as the first discharge diagnosis. The constituent ratio decreased during the 7 years (*p*<0.001), but the decrease in absolute numbers did not reach statistical significance (*p* = 0.056) (Table 1; Figures 1A,B).

### The trend of the absolute number/constituent ratio of cases of hepatobiliary diseases over time

**Figure 2:**
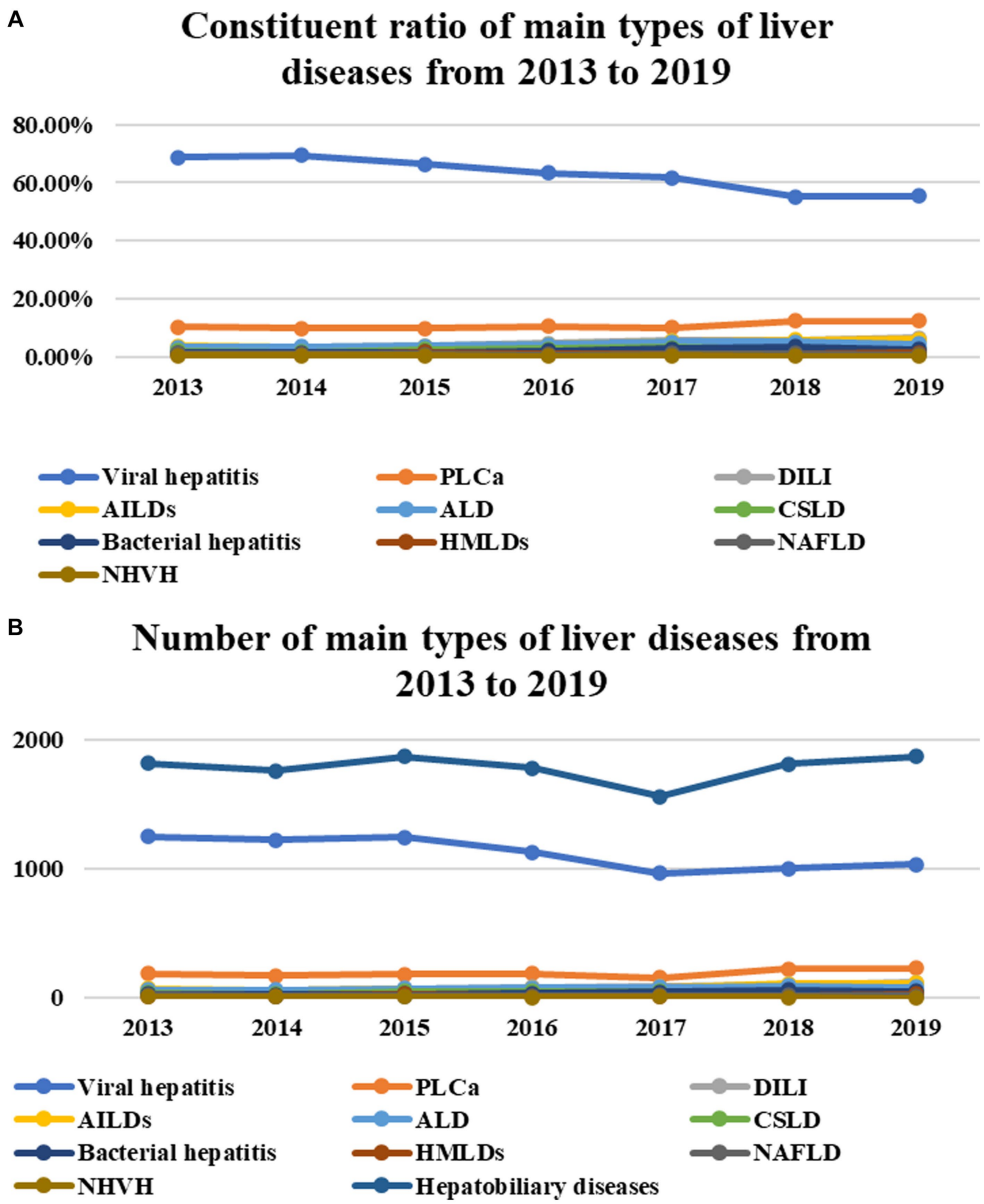
The number (proportion) of cases of viral hepatitis was decreasing year by year (*P* = 0.033, *P* < 0.001) **(A,B)**. The number and proportion of cases of PLCa did not show significant changes from 2013 to 2017 but increased significantly from 2018 to 2019 (*P* = 0.153, *P* < 0.001) **(A,B)**. The number of cases (constituent ratios) of DILI, AILDs, ALD, and bacterial hepatitis is slowly increasing year by year (*P* = 0.016, *P* < 0.001; *P* = 0.028, *P* < 0.001; *P* = 0.026, *P* < 0.001; *P* = 0.028, *P* = 0.002) **(A,B)**, while the number of cases (constituent ratios) of CSLD, HMLDs, NAFLD, and NHVH did not change significantly (*P* = 0.289, *P* = 0.070; *P* = 0.375, *P* = 0.491; *P* = 0.622, *P* = 0.791; *P* = 0.085, *P* = 0.091) **(A,B)**.

The main reason why the proportion of cases of hepatobiliary diseases to the number of total discharged cases decreased significantly was that the number (proportion) of cases of viral hepatitis was decreasing year by year, with 1,249 (69.89%), 1,222 (70.96%), 1,241 (68.19%), 1,125 (64.47%), 964 (64.22%), 1,001 (58.57%), and 1,035 (57.21%), respectively (Table 2; Figures 2A,B). The number and proportion of cases of PLCa did not show significant changes from 2013 to 2017 but increased significantly from 2018 to 2019 (*p* = 0.153, *p*<0.001). The number of cases (constituent ratios) of drug-induced liver injury (DILI), autoimmune liver diseases (AILDs), alcoholic liver disease (ALD), and bacterial hepatitis is slowly increasing year by year (*p* = 0.016, *p*<0.001; *p* = 0.028, *p*<0.001; *p* = 0.026, *p*<0.001; *p* = 0.028, *p* = 0.002) (see Table 2; Figures 2A,B), while the number of cases (constituent ratios) of chronic schistosomiasis liver disease (CSLD), hereditary metabolic liver diseases (HMLDs), non-alcoholic fatty liver disease (NAFLD), and non-hepatotropic viral hepatitis (NHVH) did not change significantly (*p* = 0.289, *p* = 0.070; *p* = 0.375, *p* = 0.491; *p* = 0.622, *p* = 0.791; *p* = 0.085, *p* = 0.091) (Table 2; Figures 2A,B).

**Table 2:** The number of discharge cases of various hepatobiliary diseases in the Department of Infectious Diseases from 2013 to 2019 and their proportion in the total number of discharge cases of hepatobiliary diseases (cases, %).

Disease categories	2013	2014	2015	2016	2017	2018	2019
Hepatobiliary diseases	1787	1722	1820	1745	1,501	1709	1806
Viral hepatitis	1,249 (69.89)	1,222 (70.96)	1,241 (68.19)	1,125 (64.47)	964 (64.22)	1,001 (58.57)	1,035 (57.21)
PLCa	188 (10.52)	170 (9.87)	181 (9.95)	185 (10.60)	155 (10.33)	222 (12.99)	230 (12.71)
DILI	52 (2.91)	57 (3.31)	68 (3.74)	83 (4.76)	85 (5.66)	102 (5.97)	120 (6.63)
AILDs	69 (3.86)	55 (3.19)	64 (3.52)	73 (4.18)	82 (5.46)	107 (6.26)	111 (6.14)
ALD	58 (3.25)	62 (3.60)	69 (3.79)	75 (4.30)	80 (5.33)	91 (5.32)	80 (4.42)
CSLD	39 (2.18)	27 (1.57)	49 (2.69)	50 (2.87)	51 (3.40)	47 (2.75)	41 (2.27)
Bacterial hepatitis	26 (1.45)	25 (1.45)	31 (1.70)	36 (2.06)	44 (2.93)	61 (3.57)	49 (2.71)
HMLDs	13 (0.73)	11 (0.64)	30 (1.65)	14 (0.80)	15 (1.00)	15 (0.88)	27 (1.49)
NAFLD	21 (1.18)	14 (0.81)	15 (0.82)	10 (0.57)	19 (1.27)	20 (1.17)	12 (0.66)
NHVH	6 (0.34)	6 (0.35)	9 (0.49)	3 (0.17)	5 (0.33)	3 (0.18)	2 (0.11)

### Trends in the absolute number/constituent ratio of cases of infectious diseases

**Figure 3:**
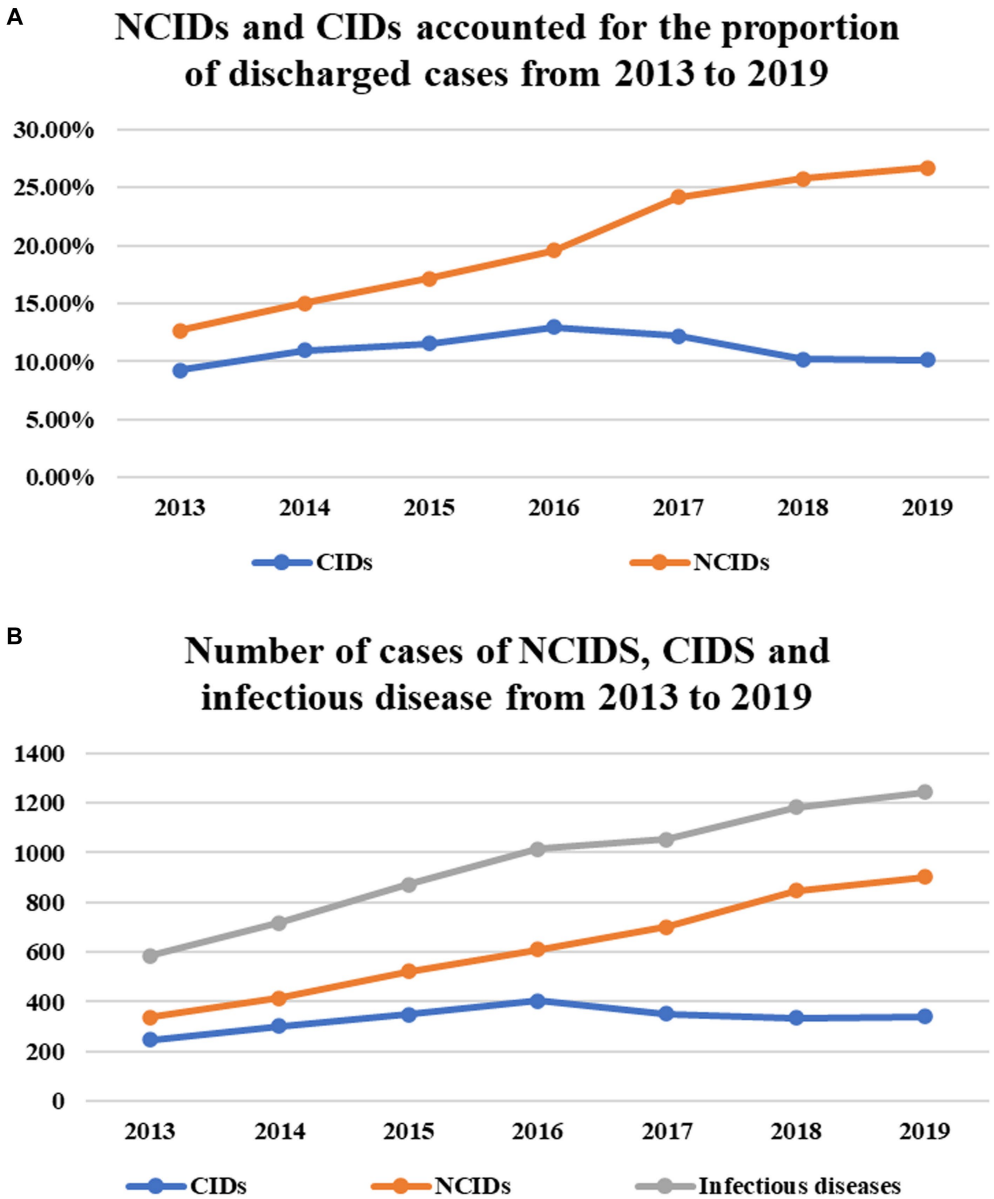
The absolute number of infectious diseases (except viral hepatitis) increased significantly (*P* = 0.015) **(B)**, and the main part of the increase was NCIDs, whose cases (constituent ratio) increased significantly year by year (*P* = 0.015, *P* < 0.001) **(A,B)**. There was no significant increase in the number of cases/constituent ratio of CIDs (*P* = 0.166, *P* = 0.480) **(A,B)**.

The absolute number and constituent ratio of infectious diseases (except viral hepatitis) increased significantly (*p* = 0.015, *p*<0.001), and the main part of the increase was NCIDs, whose cases (constituent ratio) were 340 (12.73%), 431 (15.63%), 534 (17.59%), 618 (19.80%), and 701 (24.18%), 851 (25.85%), and 918 (27.20%), respectively, increased significantly year by year (*p* = 0.015, *p*<0.001). The number of cases of CIDs (constituent ratio) were 233 (8.73%), 265 (9.61%), 322 (10.61%), 384 (12.30%), 345 (11.90%), 312 (9.48%), and 299 (8.86%), respectively, and there was no significant increase (*p* = 0.166, *p* = 0.480) (Table 1; Figures 3A,B).

**Figure 4:**
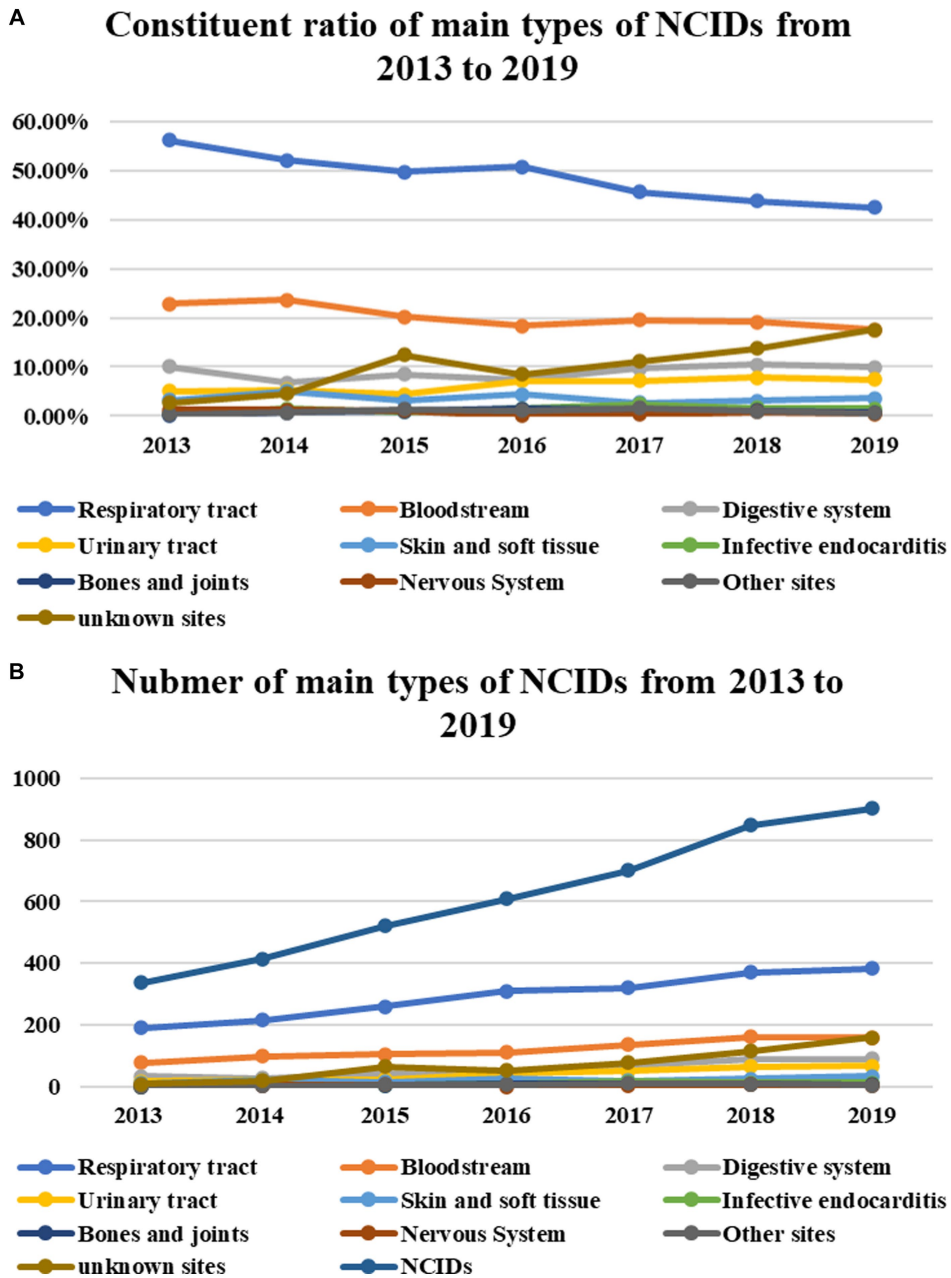
Except for infections of the nervous system, bones and joints, and other sites (*P* = 1.000, *P* = 0.065, *P* = 0.138), the number of each type of NCIDs increased significantly from year to year (Respiratory tract, *P* = 0.015; bloodstream, *P* = 0.017; digestive system, *P* = 0.020; urinary tract, *P* = 0.017; Skin and soft tissue, *P* = 0.044; Infective endocarditis, *P* = 0.030; unknown sites, *P* = 0.019), but in terms of constituent ratio, infection of respiratory system and bloodstream decreased significantly year by year (*P* < 0.001, *P* < 0.001), infections of digestive system, urinary tract, and unknown sites increased significantly year by year (*P* = 0.001, *P* < 0.001, *P* < 0.001), and the other diseases showed no obvious trend of change (skin and soft tissue infection, *P* = 0.081; infectious endocarditis, *P* = 0.394; bones and joints infections, *P* = 0.955; nervous system infections, *P* = 0.101; other sites infection, *P* = 0.728) **(A)**.

Except for infections of the nervous system, bones and joints, and other sites (*p* = 1.000, *p* = 0.065, *p* = 0.138), the absolute number of each type of NCIDs increased significantly from year to year (Respiratory infection, *p* = 0.015; bloodstream infection, *p* = 0.017; digestive system infection, *p* = 0.020; urinary tract infection, *p* = 0.017; skin and soft tissue infection, *p* = 0.044; Infective endocarditis, *p* = 0.030; infection of unknown site, *p* = 0.019). But in terms of constituent ratio, infections of the respiratory system and bloodstream decreased significantly year by year (*p*<0.001, *p*<0.001), infections of the digestive system, urinary tract, and unknown sites increased significantly year by year (*p* = 0.001, *p*<0.001, *p*<0.001), and the other diseases showed no obvious trend of change (skin and soft tissue infection, *p* = 0.081; infectious endocarditis, *p* = 0.394; bones and joints infections, *p* = 0.955; nervous system infections, *p* = 0.101; other sites infection, *p* = 0.728) (Table 3; Figures 4A,B).

**Table 3:** The number of discharge cases of various NCIDs in the Department of Infectious Diseases from 2013 to 2019 and their proportion in the total number of discharge cases of NCIDs (cases, %).

Disease categories	2013	2014	2015	2016	2017	2018	2019
NCIDs	338	415	521	610	701	847	902
Respiratory tract	190 (56.21)	216 (52.05)	259 (49.71)	310 (50.82)	320 (45.65)	371 (43.80)	383 (42.46)
Bloodstream	77 (22.78)	98 (23.61)	105 (20.15)	112 (18.36)	137 (19.54)	162 (19.13)	159 (17.63)
Digestive system	34 (10.06)	28 (6.75)	44 (8.45)	45 (7.38)	68 (9.70)	88 (10.39)	90 (9.98)
Urinary tract	17 (5.03)	22 (5.30)	23 (4.41)	44 (7.21)	50 (7.13)	66 (7.79)	67 (7.43)
Skin and soft tissue	11 (3.25)	21 (5.06)	16 (3.07)	27 (4.43)	19 (2.71)	26 (3.07)	33 (3.66)
Infective endocarditis	1 (0.30)	6 (1.45)	4 (0.77)	9 (1.48)	17 (2.43)	14 (1.65)	14 (1.55)
Bones and joints	0 (0.00)	3 (0.72)	5 (0.96)	9 (1.48)	9 (1.28)	9 (1.06)	6 (0.67)
Nervous system	4 (1.18)	5 (1.20)	6 (1.15)	0 (0.00)	2 (0.29)	7 (0.83)	4 (0.44)
Other sites	1 (0.30)	3 (0.72)	7 (1.34)	7 (1.15)	11 (1.57)	8 (0.94)	5 (0.55)
Unknown sites	9 (2.66)	19 (4.58)	65 (12.48)	52 (8.52)	78 (11.13)	116 (13.70)	159 (17.63)

**Figure 5:**
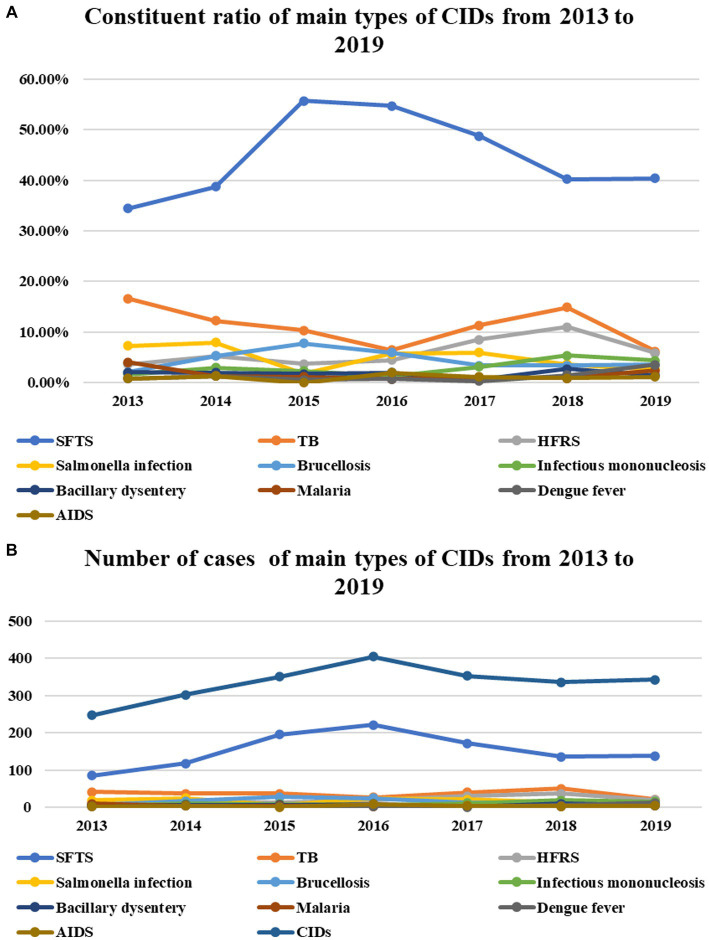
The number of cases (constituent ratio) of SFTS, which has the largest number of cases among CIDs, began to rise in 2013, peaked in 2015 and 2016, and then declined again, but the change was not statistically significant (*P* = 0.488, *P* = 0.451). While the number (constituent ratio) of TB cases from 2013–2016–2018–2019 showed a trend of decline, rise, and then decline, the change in its number did not reach statistical significance, but the decrease in its constituent ratio was statistically significant (*P* = 0.558, *P* = 0.001). The number of cases (constituent ratio) of HFRS increased slowly from 2013 to 2018 and then decreased in 2019. Similarly, the increase in its number did not reach statistical significance, but the increase in its constituent ratio was statistically significant (*P* = 0.076, *P* = 0.001). The proportion of cases of Salmonella infection has been slowly decreasing in recent years (*P* = 0.001), but the change in the number did not reach statistical significance (*P* = 0.341). The number (constituent ratio) of cases of brucellosis peaked in 2015 and then slowly decreased (*P* = 0.961, *P* = 0.028). The number (proportion) of cases of infectious mononucleosis increased from 2017 to 2019 (*P* = 0.045, *P* = 0.001). The number (constituent ratio) of cases of malaria and dengue fever increased in 2019 (*P* = 0.548, *P* = 0.056; *P* = 0.115, *P* = 0.015), while the number (constituent ratio) of AIDS cases did not change significantly in each year (*P* = 0.535, *P* = 0.992) **(A,B)**.

The number of cases (constituent ratio) of severe fever with thrombocytopenia syndrome (SFTS), which has the largest number of cases among CIDs, began to rise in 2013, peaked in 2015 and 2016, and then declined again, but the change was not statistically significant (*p* = 0.488, *p* = 0.451). While the number (constituent ratio) of Tuberculosis (TB) cases from 2013–2016 to 2018–2019 showed a trend of decline, rise, and then decline, the change in its number did not reach statistical significance, but the decrease in its constituent ratio was statistically significant (*p* = 0.558, *p*<0.001). The number of cases (constituent ratio) of hemorrhagic fever with renal syndrome (HFRS) increased slowly from 2013 to 2018 and then decreased in 2019. Similarly, the increase in its number did not reach statistical significance, but the increase in its constituent ratio was statistically significant (*p* = 0.076, *p*<0.001). The proportion of cases of salmonella infection has been slowly decreasing in recent years (*p*<0.001), but the change in the number did not reach statistical significance (*p* = 0.341). The number (constituent ratio) of cases of brucellosis peaked in 2015 and then slowly decreased (*p* = 0.961, *p* = 0.028). The number (proportion) of cases of infectious mononucleosis increased from 2017 to 2019 (*p* = 0.045, *p* = 0.001). The number (constituent ratio) of cases of malaria and dengue fever increased in 2019 (*p* = 0.548, *p* = 0.056; *p* = 0.115, *p* = 0.015), while the number (constituent ratio) of Acquired Immune Deficiency Syndrome (AIDS) cases did not change significantly in each year (*p* = 0.535, *p* = 0.992) (Table 4; Figures 5A,B).

**Table 4:** The number of discharge cases of various CIDs in the Department of Infectious Diseases from 2013 to 2019 and their proportion in the total number of discharge cases of CIDs (cases, %).

Disease categories	2013	2014	2015	2016	2017	2018	2019
CIDs	247	302	350	404	353	336	342
SFTS	85 (34.41)	117 (38.74)	195 (55.71)	221 (54.70)	172 (48.73)	135 (40.18)	138 (40.35)
TB	41 (16.60)	37 (12.25)	36 (10.29)	26 (6.44)	40 (11.33)	50 (14.88)	21 (6.14)
HFRS	9 (3.64)	16 (5.30)	13 (3.71)	18 (4.46)	30 (8.50)	37 (11.01)	20 (5.85)
Salmonella infection	18 (7.29)	24 (7.95)	6 (1.71)	23 (5.69)	21 (5.95)	12 (3.57)	9 (2.63)
Brucellosis	5 (2.02)	16 (5.30)	27 (7.71)	24 (5.94)	12 (3.40)	12 (3.57)	12 (3.51)
Infectious mononucleosis	4 (1.62)	9 (2.98)	8 (2.29)	5 (1.24)	11 (3.12)	18 (5.36)	15 (4.39)
Bacillary dysentery	5 (2.02)	6 (1.99)	6 (1.71)	8 (1.98)	2 (0.57)	9 (2.68)	5 (1.46)
Malaria	10 (4.05)	4 (1.32)	4 (1.14)	3 (0.74)	3 (0.85)	3 (0.89)	8 (2.34)
Dengue fever	2 (0.81)	4 (1.32)	2 (0.57)	3 (0.74)	1 (0.28)	5 (1.49)	12 (3.51)
AIDS	2 (0.81)	4 (1.32)	0 (0.00)	8 (1.98)	4 (1.13)	3 (0.89)	4 (1.17)

### The prevalence of the main types of NCIDs or CIDs among all discharged infectious cases during the 7-year period

**Figure 6:**
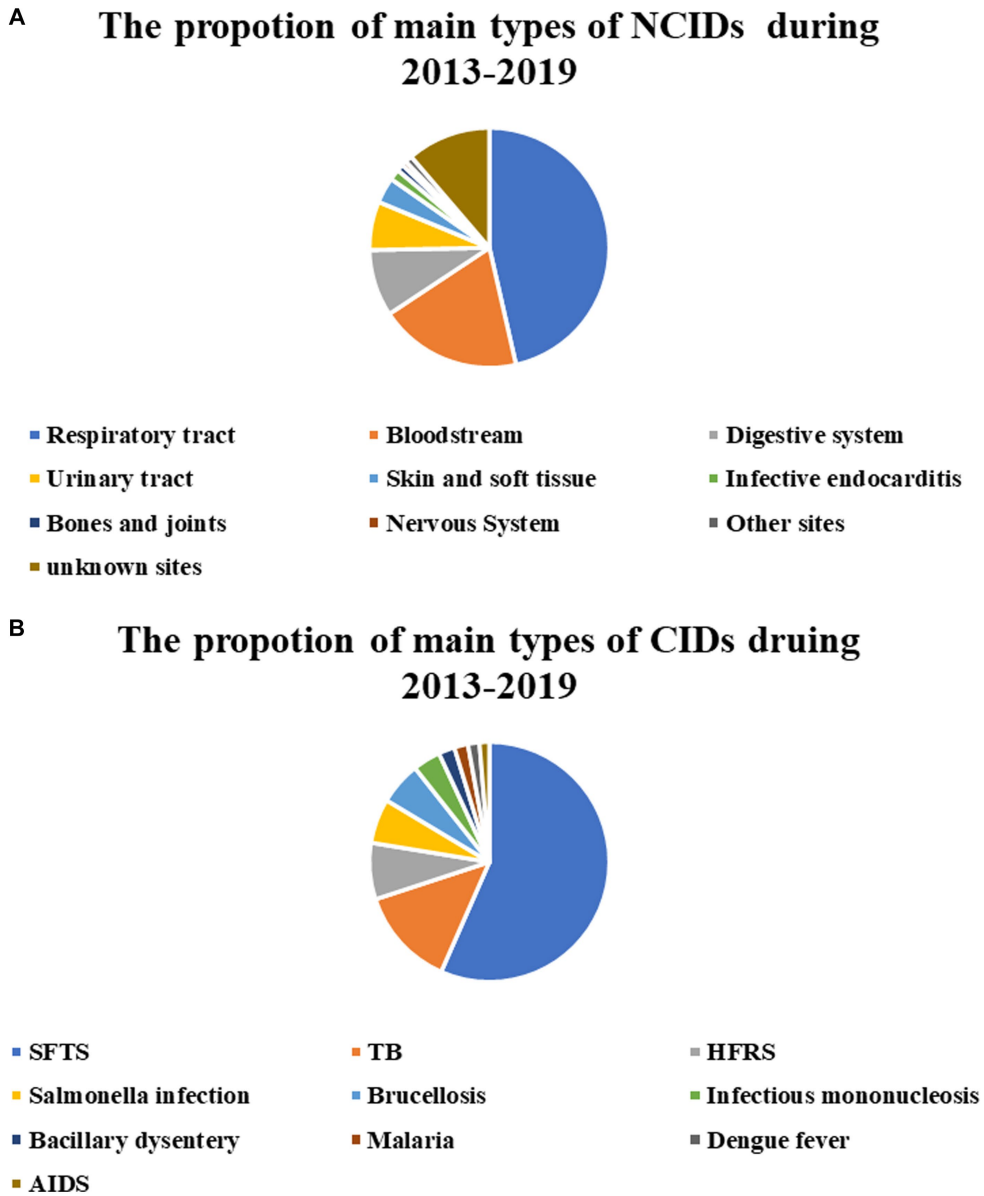
Ratio of cases of various main types of NCIDs to total cases of NCIDs **(A)**. Ratio of cases of various main types of CIDs to total cases of CIDs **(B)**.

There were 4,334 cases of NCIDs among all discharged patients during the 7-year period, and the proportion of the number of cases of infection at different sites in the total number of cases of NCIDs was as follows: Respiratory infection (47.82%), bloodstream infection (19.61%), digestive system infection (9.16%), urinary tract infection (6.67%), skin and soft tissue infections (3.53%), infectious endocarditis (1.50%), bones and joints infections (0.95%), nervous system infections (0.65%), other site infections (0.97%), and infections of unknown sites (11.49%) (Figure 6A).

There were 2,334 cases of CIDs among all discharged patients during the 7-year period, and the proportion of the number of cases of various diseases in the total number of cases of CIDs was as follows: SFTS (45.54%), TB (10.75%), HFRS (6.13%), salmonella infection (4.84%), srucellosis (4.63%), infectious mononucleosis (3.00%), bacillary dysentery (1.76%), malaria (1.50%), dengue fever (1.24%), and AIDS (1.07%) (Figure 6B).

### Top 10 CIDs for annual hospital discharge from 2013 to 2019

The number of discharged cases of various CIDs from 2013 to 2019 and their proportion in the total number of discharged cases in the same period are shown in Table 1. The major cases of CIDs discharged from the Department of Infectious Diseases during 2013–2019 were SFTS, TB, HFRS, salmonella infection, brucellosis, infectious mononucleosis, bacillary dysentery, malaria, dengue fever, and AIDS. The top 10 CIDs with the number of discharged cases from 2013 to 2019 are shown in Table 5.

**Table 5:** Top 10 CIDs in terms of annual hospital discharge from 2013 to 2019.

Sequence	2013	2014	2015	2016	2017	2018	2019
1	SFTS	SFTS	SFTS	SFTS	SFTS	SFTS	SFTS
2	TB	TB	TB	TB	TB	TB	TB
3	Salmonella infection	Salmonella infection	Brucellosis	Brucellosis	HFRS	HFRS	HFRS
4	Malaria	HFRS	HFRS	Salmonella infection	Salmonella infection	Infectious mononucleosis	Infectious mononucleosis
5	HFRS	Brucellosis	Infectious mononucleosis	HFRS	Brucellosis	Salmonella infection	Brucellosis
6	Brucellosis	Infectious mononucleosis	Salmonella infection	Bacillary dysentery	Infectious mononucleosis	Brucellosis	Dengue fever
7	Bacillary dysentery	Bacillary dysentery	Bacillary dysentery	AIDS	AIDS	Bacillary dysentery	Salmonella infection
8	Infectious mononucleosis	Malaria	Malaria	Infectious mononucleosis	Malaria	Dengue fever	Malaria
9	Dengue fever	Dengue fever	Dengue fever	Malaria	Bacillary dysentery	Malaria	Skamushi disease
10	AIDS	AIDS	AIDS	Dengue fever	Dengue fever	AIDS	Bacillary dysentery

## Discussion

In the early days of the founding of New China, infectious diseases were rampant, with a high incidence rate and widespread prevalence. Departments of infectious diseases in hospitals at all levels play an important role in the prevention and treatment of infectious diseases. In recent years, with the rapid development of the national economy, the substantial improvement of medical and health conditions, and the popularization of planned immunization, the spectrum of infectious diseases in China has undergone tremendous changes. Some long-term rampant communicable diseases have been under control, such as smallpox, which was eradicated in the early 1960s, and pestis, leishmaniasis, filariasis, malaria, and leprosy, which have been basically controlled in China. Classical communicable diseases such as neonatal tetanus, scarlet fever, measles, poliomyelitis, diphtheria, and pertussis have all been effectively controlled in China (6). With the incidence rate of communicable diseases decreasing year by year, the attention and investment of each hospital in the Department of Communicable Diseases have gradually declined, and the scale of the Department of Communicable Diseases has shrunk. Many hospitals have eliminated the Department of Communicable Diseases to varying degrees. The construction and development of the communicable disease discipline are facing severe challenges, and future development is worrying. Based on the above changes, in the past 20 years, the Department of Communicable Diseases in some traditional general hospitals has gradually changed to the Department of Infectious Diseases (3). However, we still do not know the specific situation and extent of this transformation or how the disease spectrum changes in the Department of Infectious Diseases in general hospitals after the transformation. Therefore, this paper analyzes the trend of disease constituent changes in discharged cases from the Department of infectious diseases in a large general hospital during 2013–2019 and tries to illustrate this transformation with data.

From the above results, we can see that the absolute number of discharged cases from the Department of Infectious Diseases of XX Hospital during 2013–2019 showed a gradually increasing trend, from 2,670 cases in 2013 to 3,375 cases in 2019 (*p* = 0.029). After the change of its name, the Department of Infectious Diseases is responsible for the screening, diagnosis, and treatment of various infectious diseases (including CIDs and NCIDs). The professional scope is not only limited to the diagnosis and treatment of notifiable CIDs and various emerging infectious diseases but also includes the diagnosis and treatment of some hepatobiliary diseases and NCIDs, as well as the differential diagnosis of various infectious and non-infectious diseases with fever as the main manifestation (7).

Due to the prevalence of the hepatitis B vaccine (In 2017, the coverage rate of hepatitis B vaccine for 2-year old children and 7-year old children in the province where the hospital is located reached more than 90%) (8), the strict screening of blood donors, the development of anti-hepatitis B and hepatitis C virus drugs, and the improvement of sanitary conditions, the cases of viral hepatitis are expected to gradually decrease (9). In order to capture this trend, hepatobiliary diseases were separated from other infectious diseases and discharged cases were divided into 6 categories, that is hepatobiliary diseases, infectious diseases (except viral hepatitis), neoplastic diseases (except PLCa), RCTDs, FUO, and other diseases.

Most of the diseases were hepatobiliary diseases and infectious diseases (except viral hepatitis). Accounting for 85.78–88% of all cases, the absolute number of hepatobiliary disease cases did not increase in the past 7 years or even decrease (*p* = 0.615), and its constituent ratio significantly decreased, from 66.93% in 2013 to 53.60% in 2019 (*p*<0.001). The absolute number and constituent ratio of infectious diseases (except viral hepatitis), especially NCIDs, have increased significantly (*p*<0.001), which has played a major contribution to the increase in the number of discharged cases in the Department of infectious diseases in the past 7 years. In addition, in the past 7 years, the top three CIDs admitted by the Department of Infectious Diseases of the general hospital have been SFTS, TB, and HFRS. We found that the Department of infectious diseases underwent a corresponding transformation in terms of the number of cases admitted after the name change. The absolute number and proportion of CIDs cases did not increase significantly, similar results have been found in other areas (10), while the absolute number and proportion of NCIDs cases increased significantly.

The number of cases of neoplastic diseases, RCTDs, and other diseases increases with the total number of cases per year. However, the number of cases of FUO decreased, which to some extent reflects the improvement in the diagnostic level of FUO cases in the Department of Infectious Diseases.

Consistent with our expectation, the proportion of hepatobiliary diseases in the total number of discharged patients decreased year by year (*p*<0.001), but the absolute number did not change significantly (*p* = 0.615). This indicated that the number of cases of hepatobiliary diseases did not increase with the increase in the number of discharged cases per year. We found that it was mainly due to the decline of the number and proportion of hospitalized cases of viral hepatitis, which may be due to the rapid development of anti-hepatitis B and hepatitis C drugs in the past 20 years (11, 12). Most patients with viral hepatitis can be diagnosed and treated in outpatient settings. In addition, this downward trend may also be related to fewer new cases of hepatitis B and C, which indirectly suggests that the widespread vaccination against hepatitis B and the increase in maternal and infant blocking rates, as well as strict screening of blood donors, have played a role (13). Secondly, due to the increase in the number of cases of PLCa, DILI, AILDs, ALD, and bacterial hepatitis year by year (*p* = 0.016, *p* = 0.028, *p* = 0.026, *p* = 0.028), the absolute number of hepatobiliary diseases did not decrease significantly with the decrease of the constituent ratio (*p* = 0.615, *p*<0.001).

Although the absolute numbers have risen significantly, the proportion of some NCIDs such as respiratory infection and bloodstream infection decreased significantly year by year (*p*<0.001, *p*<0.001), infections of urinary system, digestive system infection, and unknown sites increased significantly year by year (*p*<0.001, *p*<0.001, *p*<0.001). The annual variation in the numbers of cases of infectious endocarditis, skin and soft tissue infections was not statistically significant. It seems that with the improvement of medical conditions and the widespread use of antibiotics, the number of asymptomatic and occult infections is increasing.

SFTS is an emerging infectious disease caused by the SFTS virus (SFTSV). SFTS was first recognized in China in 2009 (14). It has become the number one CIDs in this general hospital in Central China. TB ranks second in the number of CIDs. The World Health Organization (WHO) estimates that TB causes about 10 million new cases and 1.5 million deaths each year, which is higher than the mortality caused by any other single infectious agent (15, 16). Since ancient times, TB has been the leading cause of disease and death in human society. While TB declined in developed countries during the 20th century, it remains a serious threat in low – and middle-income countries and emerging economies, particularly with the emergence of drug-resistant strains and associated with the human immunodeficiency virus (HIV), the treatment and control of TB remains an ongoing threat and challenge (17). HFRS is an acute infectious disease carried and transmitted by rodents. It was documented as early as 1931 in northeast China. Since then, China remains the most active HFRS endemic region (18). With the rapid development of national economy, the disease has been effectively controlled, but there are still sporadic cases in the epidemic season (19).

Over the past half-century, all aspects of many countries have been developing rapidly; people’s life expectancy has generally increased, and the aging process of the population is accelerating. Due to the aging of the body, functional decline, low immunity, and easy-to-complicate one or more chronic diseases, the risk of various infectious diseases is significantly increased in the older adult (20). In addition, in recent years, due to the gradual increase of drug-resistant bacteria and the increase in the number of people with immunodeficiency, the number of patients with infectious diseases has increased. In recent years, human beings have faced the new threat of infectious diseases. Along with economic globalization, social urbanization, and the growth and mobility of the total population, the frequent occurrence of local wars and natural disasters worsens the ecological environment, increasing the incidence frequency and transmission speed of infectious diseases and posing a huge threat to human life and health (21).

In 2019, WHO listed the top 10 threats to global health, six of which are infectious diseases (22). On the one hand, we have seen a substantial increase in both the absolute number and constituent ratio of NCIDs among discharged cases from the Department of Infectious Diseases. On November 9, 2016, the General Office of the National Health and Family Planning Commission issued “The Notice on Improving the Diagnosis and Treatment Ability of Bacterial and Fungal Infections in Secondary and Above General Hospitals,” advocating the gradual establishment of a diagnosis and treatment system for bacterial and fungal infections with the Department of Infectious Diseases as the main body. It points out the direction for the future development of the Department of Infectious Diseases at the general hospital. The Department of Infectious Diseases should strengthen its ability to diagnose and treat bacterial and fungal infections, and the focus of work should be shifted from the prevention and treatment of CIDs to the diagnosis and treatment of NCIDs such as bacterial and fungal infections (23). On the other hand, we have seen no significant reduction in the absolute number or constituent ratio of cases of CIDs. Despite the long and arduous struggle between humans and CIDs and significant achievements in the prevention and control of CIDs, so far, the only pathogenic microorganism that has been eliminated by humans is the smallpox virus. However, a variety of emerging CIDs are constantly emerging, with more and more types of CIDs, such as AIDS, SARS, influenza A H1N1, human infection of H7N9, COVID-19, and other emerging CIDs, breaking out one after another. In addition, some CIDs that had been effectively controlled have resurfaced in recent years and become prevalent again, posing a threat to human beings. For example, the incidence of TB, sexually transmitted diseases, dengue fever, schistosomiasis, diphtheria, cholera, plague, epidemic cerebrospinal meningitis, malaria, and other diseases is rising year by year (24). The US Centers for Disease Control and Prevention and World Health Organization wrote in a joint report, the proportion of people vaccinated against measles fell to 81% during the COVID-19 pandemic worldwide and still has not returned to prepandemic levels. The decline has left millions vulnerable to the virus. The total number of countries experiencing measles outbreaks now stands at 37 (25). In addition, viral hepatitis, HFRS, infectious diarrhea, and other gastrointestinal infectious diseases are still quite prevalent. Various infectious diseases have varying degrees of prevalence in different region. It can also be seen from the absolute number and constituent ratio of CIDs among the discharged cases in our department in recent years: in fact, CIDs have not decreased significantly but are showing a new epidemic trend, which is still bothering human beings and posing a serious threat to people’s lives and health (26). The pattern of long-term coexistence between humans and pathogenic microorganisms has not changed. So, the fight against infectious diseases will be a long and difficult one.

With the continuous growth of national economy, the continuous improvement of people’s cultural and health levels, and the continuous strengthening of prevention and control work. CIDs will certainly continue to be reduced, and infectious diseases will certainly continue to increase. In short, the development of infectious diseases faces both difficulties and challenges, as well as enormous opportunities.

There are three limitations to this study. (1) We come to the above conclusions on the basis of results from the single medical centre included in the study, and these conclusions need to be further validated in other hospitals in the same area or in other locations. In this study, only the most important first diagnosis of discharged cases were used as the basis for classification of diseases, but many inpatients were diagnosed with several kinds of diseases at the same time, so some combined diseases might be missed out. Due to the time limitation of the implementation of electronic medical records and the impact of the COVID-19 outbreak, this study only summarized the first discharge diagnosis during the seven-year period from 2013 to 2019, and it is difficult to analyze variations in the kinds of diseases from a longer time span.

## Conclusion

In 2013, most of the cases admitted to the Department of Infectious Diseases of the general hospital in Central China were hepatobiliary diseases, accounting for more than 68%, but the number of cases of hepatobiliary disease has gradually decreased to about 55% in 2018–2019. The absolute number and proportion of cases of infectious diseases, especially NCIDs, have increased rapidly during 2013–2019. In the future, with the popularization of prevention and treatment of viral hepatitis, the cases of viral hepatitis-related hepatobiliary diseases are expected to further decrease. Infectious diseases, especially NCIDs, are increasingly being admitted to the Department of Infectious Diseases in general hospitals in China.

## Data availability statement

The original contributions presented in the study are included in the article/supplementary material, further inquiries can be directed to the corresponding author.

## Ethics statement

The study was reviewed and approved by the ethics committee of Tongji Medical College, Huazhong University of Science and Technology. Written informed consent for participation was not required for this study in accordance with the national legislation and the institutional requirements.

## Author contributions

PY: Data curation, Formal analysis, Methodology, Writing – original draft, Writing – review & editing. LZ: Project administration, Writing – review & editing. RP: Validation, Writing – review & editing. XZ: Supervision, Writing – review & editing.

## Glossary

**Table:** 

PLCa	primary liver cancer
RCTDs	rheumatic connective tissue diseases
FUO	fever of unknown origin
NCIDs	non-communicable infectious diseases
CIDs	communicable infectious diseases
DILI	drug-induced liver injury
AILDs	autoimmune liver diseases
ALD	alcoholic liver disease
CSLD	chronic schistosomiasis liver disease
HMLDs	hereditary metabolic liver diseases
NAFLD	non-alcoholic fatty liver disease
NHVH	non-hepatotropic viral hepatitis
SFTS	severe fever with thrombocytopenia syndrome
TB	Tuberculosis
HFRS	hemorrhagic fever with renal syndrome
AIDS	acquired immune deficiency syndrome
